# (3*E*,5*E*)-3,5-Bis(4-hy­droxy-3,5-di­methoxy­benzyl­idene)oxan-4-one monohydrate

**DOI:** 10.1107/S1600536810041930

**Published:** 2010-11-27

**Authors:** Zhi-Yun Du, Hua-Rong Huang, Yu-Jun Lu, Kun Zhang, Yan-Xiong Fang

**Affiliations:** a Guangdong University of Technology, Faculty of Chemical Engineering and Light Industry, Guangzhou 510006, People’s Republic of China

## Abstract

In the title compound, C_23_H_24_O_8_·H_2_O, the six-membered ring of the oxan-4-one (tetra­hydro­pyran-4-one) ring displays an envelope conformation with the heterocyclic O atom at the flap position. The dihedral angles between the terminal benzene rings is 37.23 (10)°. Classical intermolecular O—H⋯O and weak C—H⋯O hydrogen bonds are present in the crystal structure.

## Related literature

For pharmacological activity of curcumin [systematic name (1*E*,6*E*)-1,7-bis(4-hydroxy-3-methoxyphenyl)-1,6-heptadiene-3,5-dione], see: Maheshwari *et al.* (2006 ▶). The title compound is used in the preparation of curcumin analogues, see: Du *et al.* (2006*a* ▶,*b* ▶); Liu *et al.* (2008 ▶). For a related structure, see: Abaee *et al.* (2008 ▶). For the synthesis, see: Du *et al.* (2006*a* ▶,*b* ▶); Youssef *et al.* (2004 ▶).
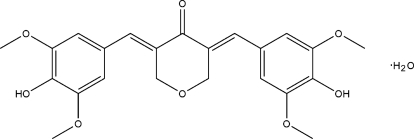


## Experimental

### 

#### Crystal data

C_23_H_24_O_8_·H_2_O
*M* *_r_* = 446.44
Monoclinic, 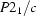

*a* = 9.203 (2) Å
*b* = 14.145 (3) Å
*c* = 17.011 (4) Å
β = 105.349 (5)°
*V* = 2135.5 (9) Å^3^
*Z* = 4
Mo *K*α radiation
μ = 0.11 mm^−1^
*T* = 293 K
0.43 × 0.40 × 0.32 mm

#### Data collection

Bruker SMART CCD 1000 area-detector diffractometer
Absorption correction: multi-scan (*SADABS*; Sheldrick, 1996 ▶) *T*_min_ = 0.955, *T*_max_ = 0.967
12756 measured reflections
4650 independent reflections
2502 reflections with *I* > 2σ(*I*)
*R*_int_ = 0.044

#### Refinement

*R*[*F* ^2^ > 2σ(*F* ^2^)] = 0.045
*wR*(*F* ^2^) = 0.116
*S* = 0.99
4650 reflections
295 parameters
H-atom parameters constrained
Δρ_max_ = 0.20 e Å^−3^
Δρ_min_ = −0.18 e Å^−3^

### 

Data collection: *SMART* (Bruker, 1999 ▶); cell refinement: *SAINT-Plus* (Bruker, 1999 ▶); data reduction: *SAINT-Plus*; program(s) used to solve structure: *SHELXTL* (Sheldrick, 2008 ▶); program(s) used to refine structure: *SHELXTL*; molecular graphics: *SHELXTL*; software used to prepare material for publication: *SHELXTL*.

## Supplementary Material

Crystal structure: contains datablocks global, I. DOI: 10.1107/S1600536810041930/xu5053sup1.cif

Structure factors: contains datablocks I. DOI: 10.1107/S1600536810041930/xu5053Isup2.hkl

Additional supplementary materials:  crystallographic information; 3D view; checkCIF report

## Figures and Tables

**Table 1:** Hydrogen-bond geometry (Å, °)

*D*—H⋯*A*	*D*—H	H⋯*A*	*D*⋯*A*	*D*—H⋯*A*
O4—H4⋯O1*W*^i^	0.82	2.06	2.833 (2)	156
O7—H7⋯O1^ii^	0.82	2.03	2.801 (2)	157
O1*W*—H1*A*⋯O7	0.86	2.38	3.162 (2)	152
O1*W*—H1*B*⋯O2^iii^	0.87	2.05	2.888 (2)	160
C13—H13⋯O7^iv^	0.93	2.54	3.390 (3)	151
C23—H23*C*⋯O1^v^	0.96	2.50	3.445 (3)	167

Symmetry codes: (i) 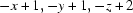; (ii) 
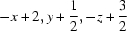
; (iii) 
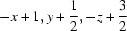
; (iv) 
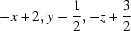
; (v) 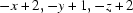.

**Fig. 1.**
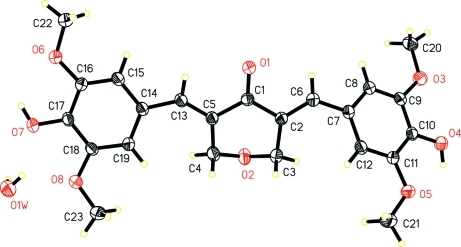
Perspective view showing 30% probability displacement ellipsoids and the atom-numbering scheme.

**Fig. 2.**
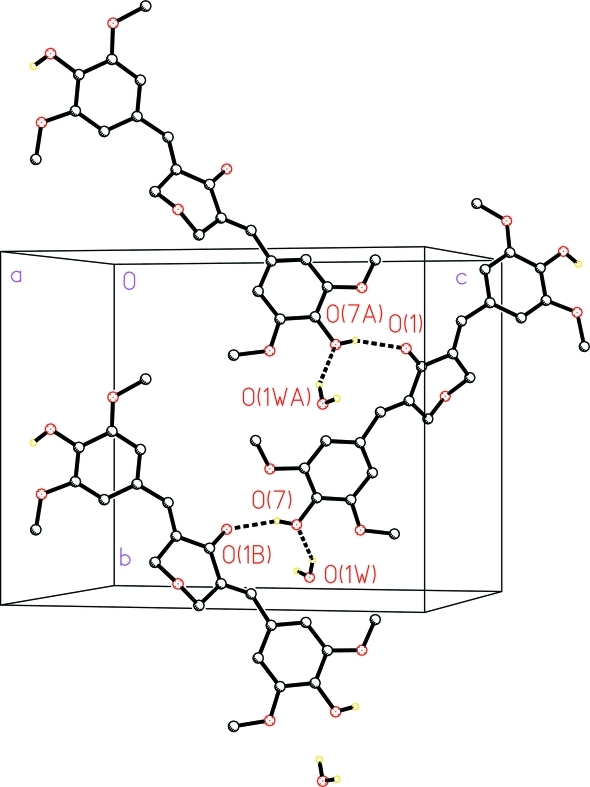
The molecules are linked via intermolecular O—H···O hydrogen bonds into one-dimensional zigzag chain along b axis. Hydrogen bonds are shown as dashed lines. Symmetry: A = -x + 2, y - 1/2, -z + 3/2; B = -x + 2, y + 1/2, -z + 3/2.

**Fig. 3.**
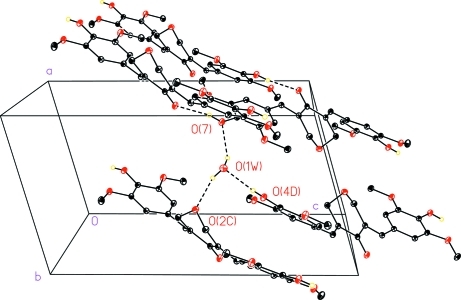
The one-dimensional zigzag chain are connected by water hydrogen bonds into a supramolecular three-dimensional complicated hydrogen bonding network. Hydrogen bonds are shown as dashed lines. Symmetry: C = -x + 1, y + 1/2, -z + 3/2; D = -x + 1, -y + 1, -z + 2.

## supplementary crystallographic information

### Comment

Curcumin possesses a wide spectrum of pharmacological activities including anti-oxidant, anti-inflammatory, antiviral, antifungal, cancer chemo preventive, cancer chemotherapeutic properties etc. (Maheshwari *et al.*, 2006). We ever screened curcumin analogues for aldose reductase (Du *et al.*, 2006a), α-glucosidase (Du *et al.*, 2006b) and thioredoxin reductase inhibition (Liu *et al.*, 2008). This class of compounds is readily synthesized by reacting a substituted benzaldehyde with tetrahydropyran-4-one; in the case of the title compound, 4-hydroxy-3,5-dimethoxybenzaldehyde was used as the reactant.

The molecular structure of the title compound contains the two 4-hydroxy-3,5-dimethoxyphenyl substituents on the tetrahydropyran-4-one, and the six-membered hetero-ring adopts an envelope conformation with the flap oxygen atom displaced by 0.682 (10) Å from the plane of the other five atoms (Figure 1).

Similar structures have been observed in the literature (Abaee *et al.*, 2008; Du *et al.*, 2006a,b).

The dihedral angles formed between the mean plane through the six atoms of the pyranone ring and two benzene rings of 4-hydroxy-3,5-dimethoxyphenyl groups are 53.86 (10) and 27.86 (10)°, the corresponding dihedral angles between two benzene rings of 4-hydroxy-3,5-dimethoxyphenyl groups is 37.23 (10) °.

In the crystal packing, the molecules are linked by intermolecular O—H···O hydrogen bonds into one-dimensional zigzag chain along *b* axis (Figure 2, table 1), and through water molecules further connecting into a supramolecular three-dimensional complicated hydrogen bonding network (Figure 3, table 1).

### Experimental

The title compound was synthesized using a general procedure (Du *et al.*, 2006a,b; Youssef *et al.*, 2004). 4-Hydroxy-3,5-dimethoxybenzy (0.01 mol) and tetrahydropyran-4-one (0.005 mol) were dissolved in THF and added 0.5 mL concentrated HCl as catalyst. The mixture was warmed at 298-303 K for 24 h, cold water was added to precipitate the yellow compound. Crystals were obtained by recrystallization from THF solution.

### Refinement

The C-bound H atoms were positioned geometrically and were included in the refinement in the riding-model approximation, with C—H = 0.96 (CH_3_), 0.97 (CH_2_) and 0.93 Å (aromatic); *U*_iso_(H) = 1.2U_eq_(C) for H atoms on secondary and tertiary C atoms, and *U*_iso_(H) = 1.5U_eq_(C) for methyl H atoms. The water and hydroxyl H atoms were located in a difference Fourier map and refined as riding with *U*_iso_(H) = 1.5U_eq_(O).

### Crystal data

**Table:**

C_23_H_24_O_8_·H_2_O	*F*(000) = 944
*M**_r_* = 446.44	*D*_x_ = 1.389 Mg m^−^^3^
Monoclinic, *P*2_1_/*c*	Mo *K*α radiation, λ = 0.71073 Å
Hall symbol: -P 2ybc	Cell parameters from 3096 reflections
*a* = 9.203 (2) Å	θ = 2.7–25.5°
*b* = 14.145 (3) Å	µ = 0.11 mm^−^^1^
*c* = 17.011 (4) Å	*T* = 293 K
β = 105.349 (5)°	Block, pale yellow
*V* = 2135.5 (9) Å^3^	0.43 × 0.40 × 0.32 mm
*Z* = 4	

### Data collection

**Table:**

Bruker SMART CCD 1000 area-detector diffractometer	4650 independent reflections
Radiation source: fine-focus sealed tube	2502 reflections with *I* > 2σ(*I*)
graphite	*R*_int_ = 0.044
φ and ω scans	θ_max_ = 27.1°, θ_min_ = 1.9°
Absorption correction: multi-scan (*SADABS*; Sheldrick, 1996)	*h* = −11→11
*T*_min_ = 0.955, *T*_max_ = 0.967	*k* = −17→18
12756 measured reflections	*l* = −12→21

### Refinement

**Table:**

Refinement on *F*^2^	Primary atom site location: structure-invariant direct methods
Least-squares matrix: full	Secondary atom site location: difference Fourier map
*R*[*F*^2^ > 2σ(*F*^2^)] = 0.045	Hydrogen site location: inferred from neighbouring sites
*wR*(*F*^2^) = 0.116	H-atom parameters constrained
*S* = 0.99	*w* = 1/[σ^2^(*F*_o_^2^) + (0.0473*P*)^2^ + 0.2822*P*] where *P* = (*F*_o_^2^ + 2*F*_c_^2^)/3
4650 reflections	(Δ/σ)_max_ < 0.001
295 parameters	Δρ_max_ = 0.20 e Å^−^^3^
0 restraints	Δρ_min_ = −0.18 e Å^−^^3^

### Special details

**Table:**

Experimental. The formulation was established by the NMR spectrum and ESI mass spectrum. ^1^H NMR (MSDO-d^6^, 300 MHz) δ (ppm): 9.03 (brs, 2H, -OH), 7.58 (s, 2H, -CH=), 6.70 (s, 4H, ArH), 4.95 (s, 4H, -CH_2_-O-CH_2_-), 3.81 (s, 12H, OCH_3_). The ESI mass spectrum showed ions at 412.
Geometry. All esds (except the esd in the dihedral angle between two l.s. planes) are estimated using the full covariance matrix. The cell esds are taken into account individually in the estimation of esds in distances, angles and torsion angles; correlations between esds in cell parameters are only used when they are defined by crystal symmetry. An approximate (isotropic) treatment of cell esds is used for estimating esds involving l.s. planes.
Refinement. Refinement of F^2^ against ALL reflections. The weighted R-factor wR and goodness of fit S are based on F^2^, conventional R-factors R are based on F, with F set to zero for negative F^2^. The threshold expression of F^2^ > 2sigma(F^2^) is used only for calculating R-factors(gt) etc. and is not relevant to the choice of reflections for refinement. R-factors based on F^2^ are statistically about twice as large as those based on F, and R- factors based on ALL data will be even larger.

### Fractional atomic coordinates and isotropic or equivalent isotropic displacement parameters (Å^2^)

**Table:**

	*x*	*y*	*z*	*U*_iso_*/*U*_eq_	
C1	0.8908 (2)	0.32659 (13)	0.97171 (12)	0.0394 (5)	
C2	0.8037 (2)	0.29050 (13)	1.02728 (12)	0.0394 (5)	
C3	0.6896 (2)	0.35663 (14)	1.04616 (13)	0.0473 (6)	
H3A	0.6147	0.3207	1.0643	0.057*	
H3B	0.7390	0.3994	1.0896	0.057*	
C4	0.7220 (2)	0.47300 (14)	0.95381 (15)	0.0498 (6)	
H4A	0.7600	0.5166	0.9985	0.060*	
H4B	0.6701	0.5096	0.9065	0.060*	
C5	0.8524 (2)	0.42155 (13)	0.93530 (12)	0.0392 (5)	
C6	0.8228 (2)	0.19984 (14)	1.05100 (13)	0.0455 (5)	
H6	0.8920	0.1658	1.0311	0.055*	
C7	0.7496 (2)	0.14758 (13)	1.10385 (13)	0.0417 (5)	
C8	0.7119 (2)	0.05293 (13)	1.08579 (14)	0.0452 (5)	
H8	0.7418	0.0233	1.0438	0.054*	
C9	0.6305 (2)	0.00271 (13)	1.12948 (13)	0.0427 (5)	
C10	0.5885 (2)	0.04587 (13)	1.19366 (13)	0.0403 (5)	
C11	0.6329 (2)	0.13891 (14)	1.21437 (13)	0.0398 (5)	
C12	0.7122 (2)	0.18956 (14)	1.16978 (13)	0.0428 (5)	
H12	0.7406	0.2517	1.1839	0.051*	
C13	0.9318 (2)	0.45266 (13)	0.88503 (13)	0.0410 (5)	
H13	1.0080	0.4116	0.8805	0.049*	
C14	0.9230 (2)	0.53765 (13)	0.83608 (13)	0.0395 (5)	
C15	0.9862 (2)	0.53228 (13)	0.77028 (13)	0.0422 (5)	
H15	1.0342	0.4771	0.7613	0.051*	
C16	0.9781 (2)	0.60806 (13)	0.71856 (13)	0.0410 (5)	
C17	0.9098 (2)	0.69190 (13)	0.73198 (13)	0.0401 (5)	
C18	0.8509 (2)	0.69876 (13)	0.79906 (13)	0.0406 (5)	
C19	0.8556 (2)	0.62257 (13)	0.85041 (13)	0.0408 (5)	
H19	0.8140	0.6276	0.8945	0.049*	
C20	0.6271 (3)	−0.13737 (15)	1.05107 (15)	0.0607 (7)	
H20A	0.5826	−0.1071	0.9998	0.091*	
H20B	0.5931	−0.2017	1.0490	0.091*	
H20C	0.7349	−0.1362	1.0616	0.091*	
C21	0.6141 (3)	0.26902 (15)	1.30040 (16)	0.0681 (7)	
H21A	0.7204	0.2816	1.3147	0.102*	
H21B	0.5747	0.2835	1.3459	0.102*	
H21C	0.5646	0.3075	1.2547	0.102*	
C22	1.1153 (3)	0.53010 (16)	0.63678 (16)	0.0637 (7)	
H22A	1.2007	0.5215	0.6829	0.096*	
H22B	1.1493	0.5405	0.5888	0.096*	
H22C	1.0530	0.4746	0.6295	0.096*	
C23	0.7318 (3)	0.79786 (15)	0.87680 (15)	0.0583 (6)	
H23A	0.6518	0.7534	0.8741	0.088*	
H23B	0.6936	0.8610	0.8768	0.088*	
H23C	0.8104	0.7875	0.9259	0.088*	
O1	0.99167 (17)	0.27831 (9)	0.95636 (9)	0.0512 (4)	
O2	0.61814 (16)	0.40948 (10)	0.97496 (10)	0.0530 (4)	
O3	0.58409 (18)	−0.08894 (9)	1.11397 (10)	0.0589 (4)	
O4	0.50522 (17)	−0.00394 (9)	1.23443 (10)	0.0535 (4)	
H4	0.4751	0.0316	1.2648	0.080*	
O5	0.58841 (17)	0.17232 (9)	1.27958 (9)	0.0524 (4)	
O6	1.03097 (17)	0.60926 (9)	0.65026 (10)	0.0564 (4)	
O7	0.89910 (18)	0.76810 (9)	0.68224 (10)	0.0527 (4)	
H7	0.9409	0.7564	0.6463	0.079*	
O8	0.79014 (17)	0.78551 (9)	0.80853 (10)	0.0547 (4)	
O1W	0.65172 (17)	0.92850 (11)	0.65729 (11)	0.0660 (5)	
H1A	0.7002	0.8780	0.6759	0.099*	
H1B	0.5849	0.9142	0.6119	0.099*	

### Atomic displacement parameters (Å^2^)

**Table:**

	*U*^11^	*U*^22^	*U*^33^	*U*^12^	*U*^13^	*U*^23^
C1	0.0492 (13)	0.0356 (11)	0.0326 (12)	−0.0025 (10)	0.0094 (11)	−0.0033 (9)
C2	0.0479 (12)	0.0356 (11)	0.0354 (13)	−0.0017 (9)	0.0120 (11)	−0.0005 (9)
C3	0.0536 (13)	0.0463 (12)	0.0442 (14)	0.0021 (10)	0.0167 (12)	0.0079 (11)
C4	0.0552 (14)	0.0403 (12)	0.0554 (15)	0.0015 (10)	0.0173 (12)	0.0078 (11)
C5	0.0464 (12)	0.0338 (11)	0.0369 (13)	−0.0033 (9)	0.0103 (11)	0.0004 (9)
C6	0.0536 (13)	0.0404 (12)	0.0456 (14)	−0.0003 (10)	0.0185 (12)	0.0013 (10)
C7	0.0470 (12)	0.0374 (11)	0.0423 (13)	0.0022 (10)	0.0149 (11)	0.0071 (10)
C8	0.0551 (13)	0.0378 (11)	0.0467 (14)	0.0045 (10)	0.0204 (12)	0.0045 (10)
C9	0.0487 (12)	0.0310 (11)	0.0483 (14)	0.0017 (9)	0.0129 (11)	0.0028 (10)
C10	0.0416 (11)	0.0368 (11)	0.0433 (13)	0.0022 (9)	0.0126 (11)	0.0078 (10)
C11	0.0445 (12)	0.0389 (11)	0.0364 (13)	0.0032 (9)	0.0111 (11)	0.0024 (10)
C12	0.0494 (12)	0.0343 (11)	0.0435 (14)	−0.0008 (9)	0.0102 (11)	0.0038 (10)
C13	0.0500 (12)	0.0321 (10)	0.0410 (13)	−0.0001 (9)	0.0121 (11)	0.0013 (10)
C14	0.0433 (11)	0.0321 (10)	0.0429 (13)	−0.0024 (9)	0.0112 (11)	0.0011 (9)
C15	0.0461 (12)	0.0323 (11)	0.0497 (14)	−0.0002 (9)	0.0155 (11)	0.0020 (10)
C16	0.0436 (12)	0.0383 (11)	0.0453 (14)	−0.0017 (9)	0.0190 (11)	0.0019 (10)
C17	0.0437 (12)	0.0323 (11)	0.0451 (14)	−0.0015 (9)	0.0133 (11)	0.0064 (10)
C18	0.0448 (12)	0.0292 (11)	0.0488 (14)	0.0020 (9)	0.0140 (11)	0.0007 (10)
C19	0.0475 (12)	0.0362 (11)	0.0415 (13)	−0.0030 (9)	0.0167 (11)	0.0008 (10)
C20	0.0782 (17)	0.0438 (13)	0.0601 (17)	0.0027 (12)	0.0184 (14)	−0.0078 (12)
C21	0.094 (2)	0.0520 (15)	0.0655 (18)	−0.0044 (13)	0.0329 (16)	−0.0146 (13)
C22	0.0762 (17)	0.0526 (14)	0.0759 (19)	0.0133 (12)	0.0440 (16)	0.0072 (13)
C23	0.0667 (15)	0.0494 (13)	0.0644 (17)	0.0141 (11)	0.0269 (14)	0.0014 (12)
O1	0.0681 (10)	0.0414 (8)	0.0521 (10)	0.0110 (7)	0.0299 (9)	0.0080 (7)
O2	0.0484 (9)	0.0532 (9)	0.0581 (10)	0.0032 (7)	0.0155 (8)	0.0173 (8)
O3	0.0842 (11)	0.0372 (8)	0.0632 (11)	−0.0119 (8)	0.0334 (10)	−0.0059 (8)
O4	0.0651 (10)	0.0424 (8)	0.0620 (12)	−0.0067 (7)	0.0328 (9)	0.0020 (8)
O5	0.0698 (10)	0.0421 (8)	0.0523 (10)	−0.0040 (7)	0.0285 (9)	−0.0043 (7)
O6	0.0751 (11)	0.0425 (8)	0.0638 (11)	0.0120 (7)	0.0399 (10)	0.0117 (8)
O7	0.0715 (11)	0.0363 (8)	0.0578 (11)	0.0075 (7)	0.0303 (9)	0.0122 (8)
O8	0.0743 (11)	0.0387 (8)	0.0598 (11)	0.0117 (7)	0.0328 (9)	0.0061 (7)
O1W	0.0629 (10)	0.0578 (10)	0.0766 (13)	−0.0020 (8)	0.0171 (9)	0.0005 (9)

### Geometric parameters (Å, °)

**Table:**

C1—O1	1.234 (2)	C15—C16	1.376 (3)
C1—C5	1.482 (3)	C15—H15	0.9300
C1—C2	1.483 (3)	C16—O6	1.374 (2)
C2—C6	1.342 (3)	C16—C17	1.389 (3)
C2—C3	1.503 (3)	C17—O7	1.358 (2)
C3—O2	1.427 (2)	C17—C18	1.390 (3)
C3—H3A	0.9700	C18—O8	1.376 (2)
C3—H3B	0.9700	C18—C19	1.381 (3)
C4—O2	1.426 (2)	C19—H19	0.9300
C4—C5	1.506 (3)	C20—O3	1.413 (3)
C4—H4A	0.9700	C20—H20A	0.9600
C4—H4B	0.9700	C20—H20B	0.9600
C5—C13	1.338 (3)	C20—H20C	0.9600
C6—C7	1.460 (3)	C21—O5	1.417 (2)
C6—H6	0.9300	C21—H21A	0.9600
C7—C12	1.390 (3)	C21—H21B	0.9600
C7—C8	1.397 (3)	C21—H21C	0.9600
C8—C9	1.383 (3)	C22—O6	1.415 (2)
C8—H8	0.9300	C22—H22A	0.9600
C9—O3	1.369 (2)	C22—H22B	0.9600
C9—C10	1.392 (3)	C22—H22C	0.9600
C10—O4	1.358 (2)	C23—O8	1.413 (3)
C10—C11	1.395 (3)	C23—H23A	0.9600
C11—O5	1.365 (2)	C23—H23B	0.9600
C11—C12	1.384 (3)	C23—H23C	0.9600
C12—H12	0.9300	O4—H4	0.8200
C13—C14	1.452 (3)	O7—H7	0.8200
C13—H13	0.9300	O1W—H1A	0.8572
C14—C15	1.393 (3)	O1W—H1B	0.8743
C14—C19	1.403 (3)		
			
O1—C1—C5	121.49 (18)	C16—C15—C14	120.50 (18)
O1—C1—C2	120.54 (17)	C16—C15—H15	119.8
C5—C1—C2	117.97 (18)	C14—C15—H15	119.8
C6—C2—C1	118.06 (18)	O6—C16—C15	125.38 (18)
C6—C2—C3	125.04 (19)	O6—C16—C17	113.92 (17)
C1—C2—C3	116.64 (17)	C15—C16—C17	120.70 (19)
O2—C3—C2	109.61 (17)	O7—C17—C16	122.48 (19)
O2—C3—H3A	109.7	O7—C17—C18	118.44 (17)
C2—C3—H3A	109.7	C16—C17—C18	119.08 (18)
O2—C3—H3B	109.7	O8—C18—C19	124.58 (19)
C2—C3—H3B	109.7	O8—C18—C17	114.67 (17)
H3A—C3—H3B	108.2	C19—C18—C17	120.75 (18)
O2—C4—C5	111.89 (16)	C18—C19—C14	119.97 (19)
O2—C4—H4A	109.2	C18—C19—H19	120.0
C5—C4—H4A	109.2	C14—C19—H19	120.0
O2—C4—H4B	109.2	O3—C20—H20A	109.5
C5—C4—H4B	109.2	O3—C20—H20B	109.5
H4A—C4—H4B	107.9	H20A—C20—H20B	109.5
C13—C5—C1	117.08 (18)	O3—C20—H20C	109.5
C13—C5—C4	125.08 (18)	H20A—C20—H20C	109.5
C1—C5—C4	117.74 (17)	H20B—C20—H20C	109.5
C2—C6—C7	128.60 (19)	O5—C21—H21A	109.5
C2—C6—H6	115.7	O5—C21—H21B	109.5
C7—C6—H6	115.7	H21A—C21—H21B	109.5
C12—C7—C8	119.13 (19)	O5—C21—H21C	109.5
C12—C7—C6	122.26 (18)	H21A—C21—H21C	109.5
C8—C7—C6	118.58 (19)	H21B—C21—H21C	109.5
C9—C8—C7	120.8 (2)	O6—C22—H22A	109.5
C9—C8—H8	119.6	O6—C22—H22B	109.5
C7—C8—H8	119.6	H22A—C22—H22B	109.5
O3—C9—C8	124.67 (19)	O6—C22—H22C	109.5
O3—C9—C10	115.41 (18)	H22A—C22—H22C	109.5
C8—C9—C10	119.92 (18)	H22B—C22—H22C	109.5
O4—C10—C9	118.97 (18)	O8—C23—H23A	109.5
O4—C10—C11	121.84 (19)	O8—C23—H23B	109.5
C9—C10—C11	119.19 (19)	H23A—C23—H23B	109.5
O5—C11—C12	125.57 (18)	O8—C23—H23C	109.5
O5—C11—C10	113.63 (17)	H23A—C23—H23C	109.5
C12—C11—C10	120.8 (2)	H23B—C23—H23C	109.5
C11—C12—C7	120.00 (19)	C4—O2—C3	110.80 (16)
C11—C12—H12	120.0	C9—O3—C20	118.01 (17)
C7—C12—H12	120.0	C10—O4—H4	109.5
C5—C13—C14	132.99 (19)	C11—O5—C21	118.12 (17)
C5—C13—H13	113.5	C16—O6—C22	117.36 (16)
C14—C13—H13	113.5	C17—O7—H7	109.5
C15—C14—C19	118.95 (18)	C18—O8—C23	117.46 (16)
C15—C14—C13	116.33 (18)	H1A—O1W—H1B	108.0
C19—C14—C13	124.71 (19)		
			
O1—C1—C2—C6	−9.1 (3)	C6—C7—C12—C11	175.20 (19)
C5—C1—C2—C6	170.58 (19)	C1—C5—C13—C14	176.8 (2)
O1—C1—C2—C3	176.48 (19)	C4—C5—C13—C14	0.4 (4)
C5—C1—C2—C3	−3.8 (3)	C5—C13—C14—C15	−158.0 (2)
C6—C2—C3—O2	−136.3 (2)	C5—C13—C14—C19	21.6 (4)
C1—C2—C3—O2	37.7 (2)	C19—C14—C15—C16	−2.2 (3)
O1—C1—C5—C13	0.0 (3)	C13—C14—C15—C16	177.47 (18)
C2—C1—C5—C13	−179.67 (18)	C14—C15—C16—O6	−177.28 (19)
O1—C1—C5—C4	176.63 (19)	C14—C15—C16—C17	1.5 (3)
C2—C1—C5—C4	−3.1 (3)	O6—C16—C17—O7	−0.9 (3)
O2—C4—C5—C13	152.0 (2)	C15—C16—C17—O7	−179.78 (19)
O2—C4—C5—C1	−24.3 (3)	O6—C16—C17—C18	179.54 (18)
C1—C2—C6—C7	−179.0 (2)	C15—C16—C17—C18	0.7 (3)
C3—C2—C6—C7	−5.1 (4)	O7—C17—C18—O8	−1.6 (3)
C2—C6—C7—C12	−35.3 (3)	C16—C17—C18—O8	178.00 (18)
C2—C6—C7—C8	142.7 (2)	O7—C17—C18—C19	178.38 (19)
C12—C7—C8—C9	3.9 (3)	C16—C17—C18—C19	−2.0 (3)
C6—C7—C8—C9	−174.22 (19)	O8—C18—C19—C14	−178.75 (18)
C7—C8—C9—O3	177.48 (19)	C17—C18—C19—C14	1.3 (3)
C7—C8—C9—C10	−1.6 (3)	C15—C14—C19—C18	0.8 (3)
O3—C9—C10—O4	−0.8 (3)	C13—C14—C19—C18	−178.80 (19)
C8—C9—C10—O4	178.29 (19)	C5—C4—O2—C3	60.9 (2)
O3—C9—C10—C11	179.12 (18)	C2—C3—O2—C4	−67.7 (2)
C8—C9—C10—C11	−1.7 (3)	C8—C9—O3—C20	2.7 (3)
O4—C10—C11—O5	1.7 (3)	C10—C9—O3—C20	−178.19 (19)
C9—C10—C11—O5	−178.28 (18)	C12—C11—O5—C21	5.3 (3)
O4—C10—C11—C12	−177.24 (19)	C10—C11—O5—C21	−173.58 (19)
C9—C10—C11—C12	2.8 (3)	C15—C16—O6—C22	−7.4 (3)
O5—C11—C12—C7	−179.31 (18)	C17—C16—O6—C22	173.82 (19)
C10—C11—C12—C7	−0.5 (3)	C19—C18—O8—C23	1.6 (3)
C8—C7—C12—C11	−2.8 (3)	C17—C18—O8—C23	−178.42 (19)

### Hydrogen-bond geometry (Å, °)

**Table:**

*D*—H···*A*	*D*—H	H···*A*	*D*···*A*	*D*—H···*A*
O4—H4···O1W^i^	0.82	2.06	2.833 (2)	156
O7—H7···O1^ii^	0.82	2.03	2.801 (2)	157
O1W—H1A···O7	0.86	2.38	3.162 (2)	152
O1W—H1B···O2^iii^	0.87	2.05	2.888 (2)	160
C13—H13···O7^iv^	0.93	2.54	3.390 (3)	151
C23—H23C···O1^v^	0.96	2.50	3.445 (3)	167

Symmetry codes: (i) −*x*+1, −*y*+1, −*z*+2; (ii) −*x*+2, *y*+1/2, −*z*+3/2; (iii) −*x*+1, *y*+1/2, −*z*+3/2; (iv) −*x*+2, *y*−1/2, −*z*+3/2; (v) −*x*+2, −*y*+1, −*z*+2.
